# Modulation of social valence by insular cortex activity during acute social isolation in mice

**DOI:** 10.1186/s13041-025-01236-4

**Published:** 2025-07-15

**Authors:** Kanae Hiyoshi, Daichi Matsushita, Ayako M. Watabe

**Affiliations:** Institute of Clinical Medicine and Research, Research Center for Medical Sciences, The Jikei University School of Medicine, 163-1 Kashiwashita, Kashiwa, Chiba, 277-8567 Japan

**Keywords:** Short-term social isolation, Insular cortex, Rebound social interaction

## Abstract

For social animals, social isolation is a potential threat to survival, and therefore can be considered innately aversive. Long-term social isolation induces a variety of social and affective deficits and has been used as a stress model in animal studies, with increasing insight into its underlying neural mechanisms. In contrast, short-term social isolation is known to elicit prosocial behaviors such as rebound social interactions, yet the neural basis of these adaptive responses remains poorly understood. Here, we investigated the effects of short-term social isolation on social and appetitive behaviors and examined the role of the insular cortex in modulating social preference in male mice. Three days of social isolation increased social contacts in a three-chamber social preference test. Additionally, socially isolated mice showed higher food intake in the home cage compared with the group-housed mice, and those exhibiting a higher social preference following social isolation also tended to consume more food during the isolation, postulating a potential correlation of social craving and food craving. Furthermore, chemogenetic suppression of the insular cortex during social isolation reduced rebound social interactions. We propose that the insular cortex modulates social valence by serving as an alert center for social deprivation. Our findings may help advance understanding of the neuronal mechanisms that underlie adaptive social and appetitive behaviors in response to social isolation.

**Supplementary Information:**

The online version contains supplementary material available at 10.1186/s13041-025-01236-4.

## Introduction

Detecting potential harm in the environment and promoting appropriate adaptive behaviors are crucial for animal survival. For social animals, social isolation poses a potential threat for survival and therefore is considered innately aversive. Indeed, social isolation is a significant risk factor for morbidity and mortality [1–3]. In animal models, long-term social isolation is widely used as a stress model, which exhibits a variety of social and affective deficits, such as increased aggression, social avoidance, and enhanced fear responses in various species, including flies, rodents, and humans [4–7]. In contrast, short-term social isolation leads to different behavioral outcomes from those of long-term social isolation and can even promote affiliative social behaviors [8]. Such behavioral differences can be interpreted as adaptive responses to restore social connection, aligning with the concept of social homeostasis, which refers to the maintenance of stable social interactions despite environmental fluctuations [9]. However, compared to long-term social isolation, the neural mechanisms underlying behavioral changes in response to short-term isolation remain only partially understood.

The insular cortex has extensive connections with cortical, subcortical, and brainstem regions and is involved in sensory and emotional processing of interoceptive and exteroceptive signals, including pain and taste as well as physiological states such as hunger [10–12]. Furthermore, the insular cortex projects to the limbic system, including the amygdala, and contributes to the modulation of emotional behaviors. For instance, the insular cortex receives bodily feedback signals and bidirectionally regulates anxiety-related behaviors and fear extinction, and these findings highlight its role as a regulator of emotional homeostasis [13, 14]. Considering the anatomical advantages and these functional findings of the insular cortex, it is plausible that the insular cortex also modulates socially adaptive behaviors in response to socially aversive situations.

Recent studies have implicated the insular cortex in stress-related social and anxiety disorders, as well as social recognition and social decision-making [15–17]. One of the recent key reports on the involvement of the insular cortex in social isolation found that some insular cortex neurons, which project inter-hemispherically to the contralateral insula cortex, became more excitable after short-term social isolation compared to group-housed conditions [18]. Moreover, the ablation of these insular neurons impaired social preference following short-term social isolation, whereas their inhibition only during the social preference test had no effect on social preference. While these findings suggest that insular cortex activation during isolation is crucial for rebound social interaction following short-term isolation, further validation about its necessity is still required. Here, we investigated whether insular cortex activity during short-term social isolation is required for the modulation of subsequent social preference.

## Materials and methods

### Animals

All the experimental protocols in this study including the use of animals were approved by the Institutional Animal Care and Use Committee of the Jikei University (Tokyo, Japan; Approval No. 2018-072, 2019-045, 2024-056). All experiments complied with the Guidelines for Proper Conduct of Animal Experiments by the Science Council of Japan (2006) and those recommended by the International Association for the Study of Pain. All efforts were made to reduce the number of animals used and the suffering of the animals. Male C57BL/6JJmsSlc mice (Japan SLC, Shizuoka, Japan) were group-housed with 4–5 mice per cage, except during the period of social isolation, and were provided with food and water *ad libitum* on a 12 h light/dark cycle (lights on at 07:00 and off at 19:00).

### Social isolation

Male mice aged 8–12 weeks were used in our behavioral assay. Mice were initially group-housed and then randomly assigned to two different rearing condition groups: social isolation and group-housing. For short-term social isolation, mice were placed in a home-cage with new bedding (1 animal per cage) and underwent social isolation for 3 days before the behavioral tests began. Group-housing mice were also transferred to a home cage with new bedding and reared for 3 days under group housing conditions (4–5 animals per cage). Each rearing condition was maintained until all behavioral experiments were completed. In addition, food intake per cage and body weight of each animal were measured before and 3 days after group-housing or isolation treatment. To compare the food intake of isolated mice with that of group-housed mice, the total food intake per cage was divided by the number of mice per cage to obtain the average food intake per group-housed mouse. Then, for each experimental batch (isolation cages: 4–5, group housing cage: 1), the ratio of food intake in each isolated mouse to the average food intake in group-housed mice was calculated. To assess the effects of short-term social isolation on food intake and body weight, 13 animals per group were used (Grouped, *n* = 13 (3 cages); Isolated, *n* = 13). To examine the correlation between food intake and social preference in isolated mice, 8 animals were used (Isolated, *n* = 8).

### Surgical procedures for adeno-associated virus (AAV) microinjection

Five-week-old male mice were intraperitoneally anesthetized with a mixture of medetomidine hydrochloride (0.75 mg/kg; Zenoaq, Fukushima), midazolam (4.0 mg/kg; Astellas, Tokyo, Japan), and butorphanol tartrate (5.0 mg/kg; Meiji Seika Pharma, Tokyo, Japan) and fixed in a stereotaxic device. AAV5-hSyn-hM4D(Gi)-mCherry (purchased from Addgene), AAVDJ-Syn-mCherry (a generous gift from Prof. Toshihisa Ohtsuka, University of Yamanashi) and AAVDJ-Syn-EYFP (a generous gift from Prof. Toshihisa Ohtsuka, University of Yamanashi) were used for microinjection. AAV vectors (0.3 µl) were microinjected bilaterally into the insular cortex (0.7 mm posterior to bregma, 4.0 mm lateral to midline, and 4.1 mm ventral to the skull surface at bregma) using a Hamilton microsyringe (1701RN Neuros Syringe, 33 G, 10 µl; Hamilton Company, Reno, NV, USA), as previously described [19]. The injection speed (50 nl/minute) was controlled by a microsyringe pump (UMP3; UltraMicroPumpII with SYS-Micro4 Controller, UMP2, UMC4; World Precision Instruments, Sarasota, FL). Injection syringes were left in place for 10 min before withdrawing. Expression in the insular cortex was confirmed for all animals used in this study.

### Open-field test

An open-field test was conducted using a square apparatus (100 lx) with grey walls (50 cm width × 50 cm length × 30 cm height, OF-3002 F; O’Hara & Co., Ltd.). Mice were placed in the corner of the apparatus and allowed to explore freely for 10 min. Ambulation was recorded (2 fps) and analyzed using a video-computerized tracking system (TimeOFCR1; O’Hara & Co., Ltd.). The entire open-field area was divided into 25 (5 × 5) squares, and the central nine (3 × 3) squares surrounded by the peripheral 16 squares were considered the central region. The total travelled distance and time spent in the central region were analyzed as proxies for basic locomotor activity and general anxiety levels, respectively. Open-field test was performed using 8 animals per group (Grouped, *n* = 8; Isolated, *n* = 8).

### Social preference test (SPT)

The social preference test was conducted in a plexiglass three-chambered arena (62 cm width × 40 cm length × 22 cm height, CSI-4012 C; O’Hara & Co., Ltd.) in a sound attenuating box. For habituation to the arena, the test mouse was placed in the middle chamber and allowed to freely explore for 10 min under 20 lx illumination conditions, with empty cages placed in the corners of each side chamber. After the habituation session, the test mouse was temporarily removed from the arena, and an unfamiliar mouse (male, aged 0–2 weeks younger than the test mouse) was placed in one cage while the other cage was left empty. The test mouse was then returned to the central chamber, and the 5 min test session was started, allowing free exploration. Mouse behavior was captured using a digital camera at 25 frames per second and the time spent around each cage was calculated using Time CSI software (O’Hara & Co., Ltd.t). Social preference score was calculated as (time spent around the social-cue cage (%) - time spent around the empty cage (%)) during the first 0–2 min of the SPT. To assess the effects of short-term social isolation on social preference, 8 animals per group were used (Grouped, *n* = 8; Isolated, *n* = 8). To examine the effects of the inhibition of the insular cortex in group-housed mice and isolated mice, 4–6 animals were used per group (Group-housed: Ctrl, *n* = 4; hM4Di, *n* = 5, Isolated: Ctrl, *n* = 6; hM4Di, *n* = 6).

### Chemogenetic manipulations

For chemogenetic inhibition of insular cortex, the Designer Receptors Exclusively Activated by Designer Drugs (DREADD) agonist clozapine N-oxide (CNO) (Sigma Aldrich) was diluted in saline to 0.5 mg/mL and intraperitoneally administered to the mice at 1 mg/kg. CNO was administered twice daily at 09:00 and 18:00 for 3 days during the period of social isolation. The SPT after 3 days of social isolation was performed at least 4 h after the last CNO administration.

### Statistical analysis

Statistical analyses were performed using OriginPro software (LightStone). Differences were considered statistically significant at *p* < 0.05. Graphs were created using OriginPro. See also Additional file 1.

## Results

### Short-term social isolation increased social interaction

**Fig. 1:**
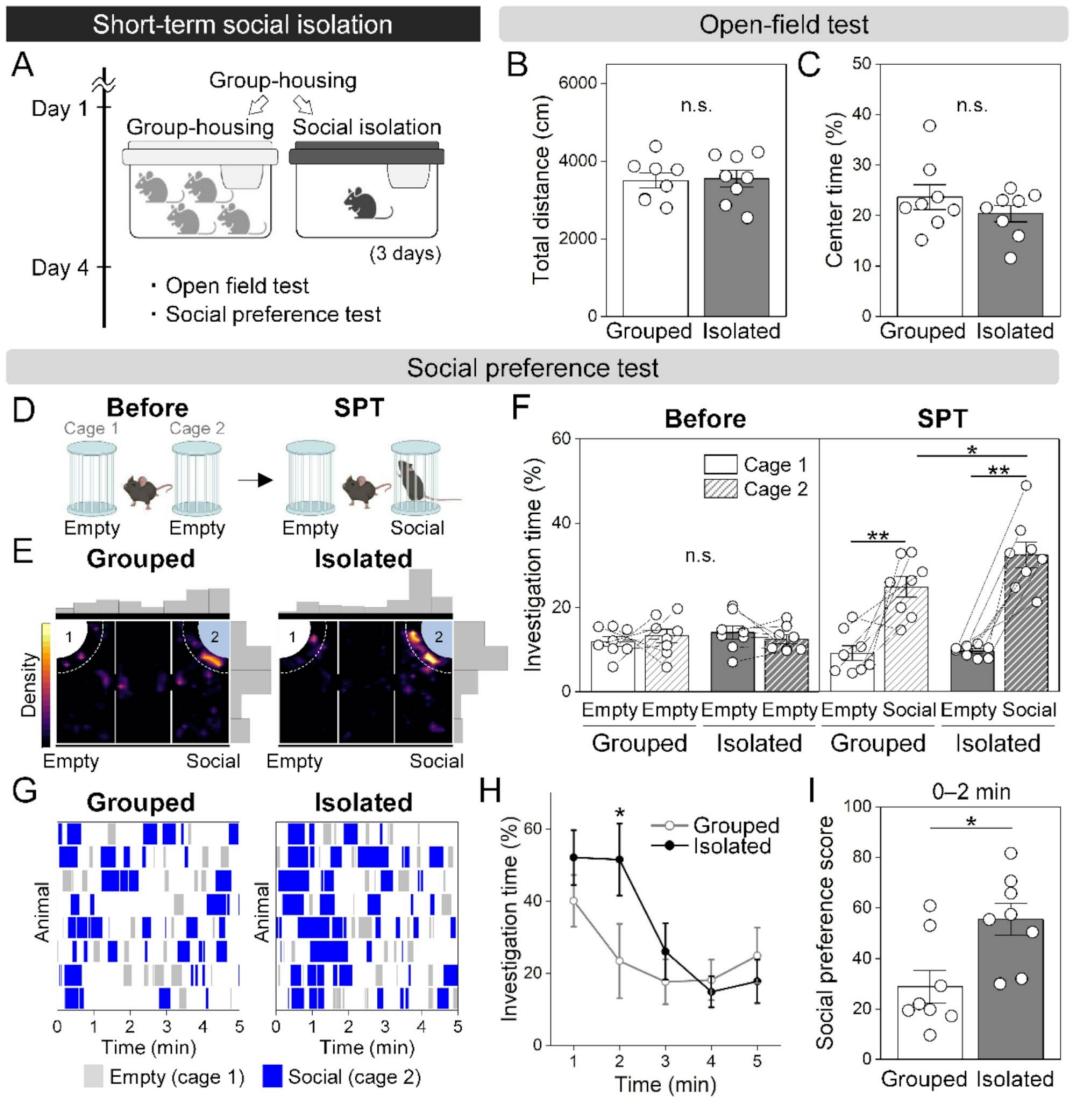
Changes in basal locomotor activity and social behaviors following short-term social isolation. (**A**) Experimental schedule to assess the effects of short-term social isolation on social and affective behaviors. (**B**, **C**) Total distance traveled (**B**) and time spent in the center region (**C**) during the open-field test. (**D**) Schematic diagram of the social preference test (SPT). A 10-minute session of presenting empty cages (Before) was performed prior to the SPT for equipment habituation. (**E**) Representative density heatmaps showing time spent in the SPT for 5 min. (**F**) Time spent around the cage during the habituation (Before) and the SPT session. (**G**, **H**) Dynamics of social interaction in the SPT. Time course of stays around the cage in each mouse are shown in (**G**) (gray: empty cage; blue: social-cue cage), and temporal changes in time spent around the social-cue cage are shown in (**H**). (**I**) Social preference score calculated for 0–2 min in the SPT. Grouped, *n* = 8; Isolated, *n* = 8; two-tailed unpaired *t*-test (**B**, **C**, **I**); two-way repeated measures ANOVA followed by Tukey’s post hoc test (**F**, **H**); *p < 0.05, **p < 0.01. Data are presented as mean ± S.E.M

To examine how short-term social isolation affects social and affective behaviors, we first conducted the open-field test, which assessed basic locomotor activity and general anxiety levels, following 3 days of isolation (Fig. 1A). There were no significant differences in the total traveled distance and the time spent in the center region between socially isolated mice and group-housed mice (Fig. 1B, C, two-tailed unpaired *t*-test). These results are consistent with previously reported findings on 24-hour social isolation [20].

To investigate whether short-term social isolation affects social preference, we performed three-chamber social preference test (SPT) (Fig. 1D). Although no significant preference was observed between two empty cages during the 10-minute session prior to the SPT, socially isolated mice spent more time around the social-cue cage containing an unfamiliar mouse than group-housed mice in the SPT (Fig. 1E, F, two-way repeated measures ANOVA followed by Tukey’s post hoc test). Interestingly, isolated mice appeared to be more strongly attracted to the social-cue cage, particularly during the early phase of the SPT (Fig. 1G, H, two-way repeated measures ANOVA followed by Tukey’s post hoc test). Indeed, the social preference score during the 0–2 min of the SPT was significantly higher in isolated mice compared to group-housed mice (Fig. 1I, two-tailed unpaired *t*-test). These results indicate that 3 days of short-term social isolation induces rebound social interaction, as reported previously [8], and suggest that this enhanced social interaction is particularly prominent immediately after encountering an unfamiliar mouse.

### Increased food consumption in socially isolated mice

**Fig. 2:**
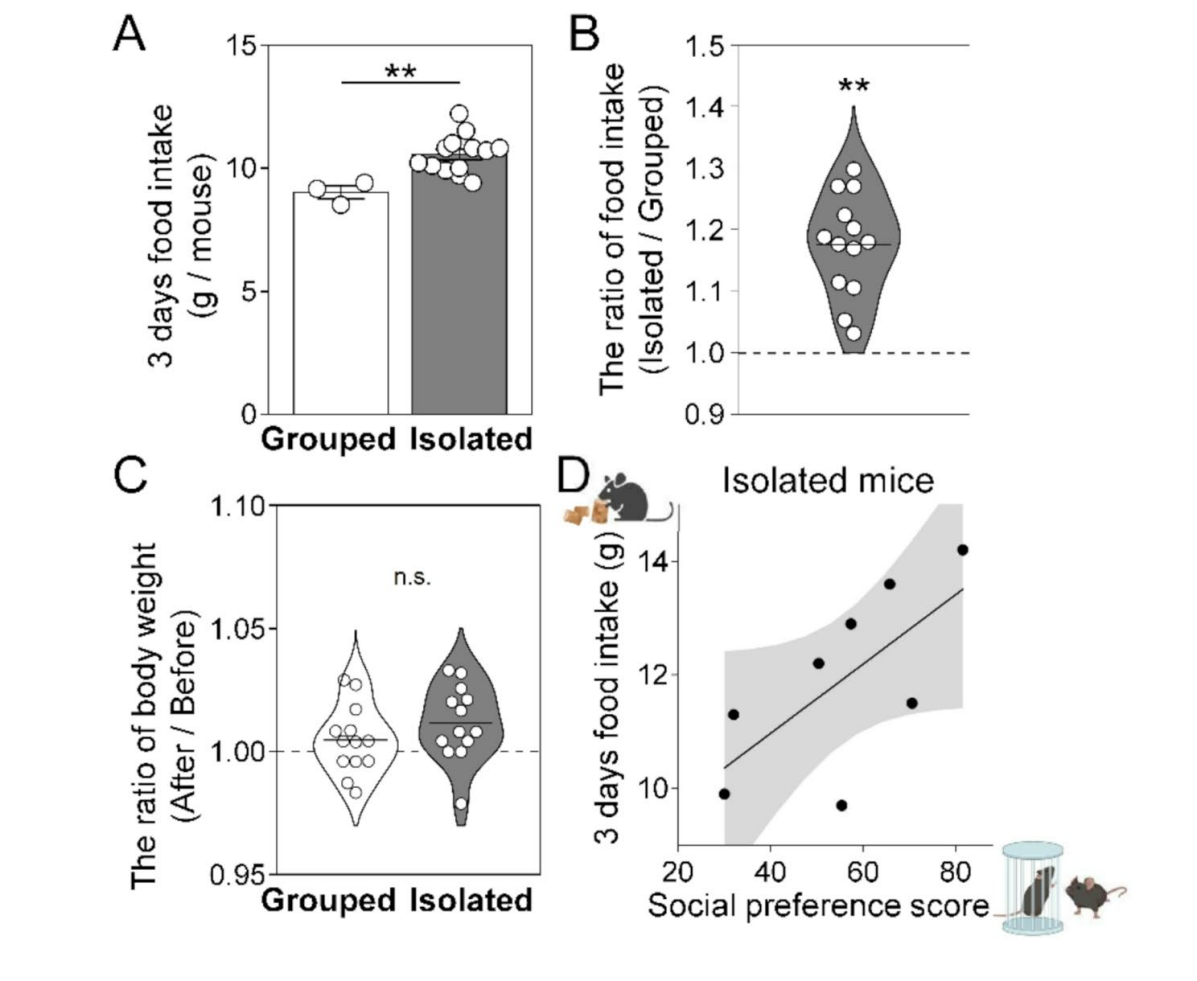
Short-term social isolation increased food consumption in the home cage. (**A**) Food intake per mouse for 3 days (Grouped, *n* = 3 cages (13 mice); Isolated, n =: 13 cages (13 mice); two-tailed unpaired *t*-test). (**B**) The ratio of food intake in the home cage of isolated mice to that of group-housed mice. The food intake for 3 days of each isolated mouse was divided by the average food intake per group-housed mouse. Grouped, *n* = 13 mice (3 cages); Isolated, n=: 13 mice; one-sample *t*-test; **p < 0.01 (**C**) Change in body weight over 3 days, standardized by the weight before isolation or group-housing treatment. Grouped, *n* = 13; Isolated, *n* = 13; two-tailed unpaired *t*-test. (**D**) Correlation between 3-day food intake and social preference score in socially isolated mice (*n* = 8). Regression lines with 95% confidence intervals were presented, and Pearson’s correlation was used for analysis (*r* = 0.67054, *p* = 0.06877). Data are presented as mean ± S.E.M

As mentioned above, isolation-induced changes in social behavior have been observed.　Assuming that social isolation induces an aversive state, we hypothesized that additional affective behavioral changes might occur during isolation. To explore this, we focused on food consumption in the home-cage during the isolation period. Interestingly, the food intake per individual during the isolation period was higher in isolated mice compared to group-housed mice (Fig. 2A). This was also confirmed by normalizing food intake, using the ratio of the 3-day intake in socially isolated mice to the average intake per mouse in group housing (Fig. 2B, one-sample *t*-test), suggesting that short-term social isolation induces overconsumption of food in the home-cage. In contrast, there was no significant difference in body weight change over 3 days between group housing and social isolation (Fig. 2C, two-tailed unpaired *t*-test), suggesting that the increased food intake may not have been sufficient to cause detectable changes in body weight during the short-term isolation period. Notably, the amount of food consumed during isolation tended to correlate with the level of social preference following isolation (Fig. 2D; Pearson correlation coefficient: *r* = 0.67054, *p* = 0.06877), suggesting that food consumption during isolation may be associated with a form of social craving.

### Chemogenetic inhibition of insular cortex during social isolation suppressed the enhancement of social preference

**Fig. 3:**
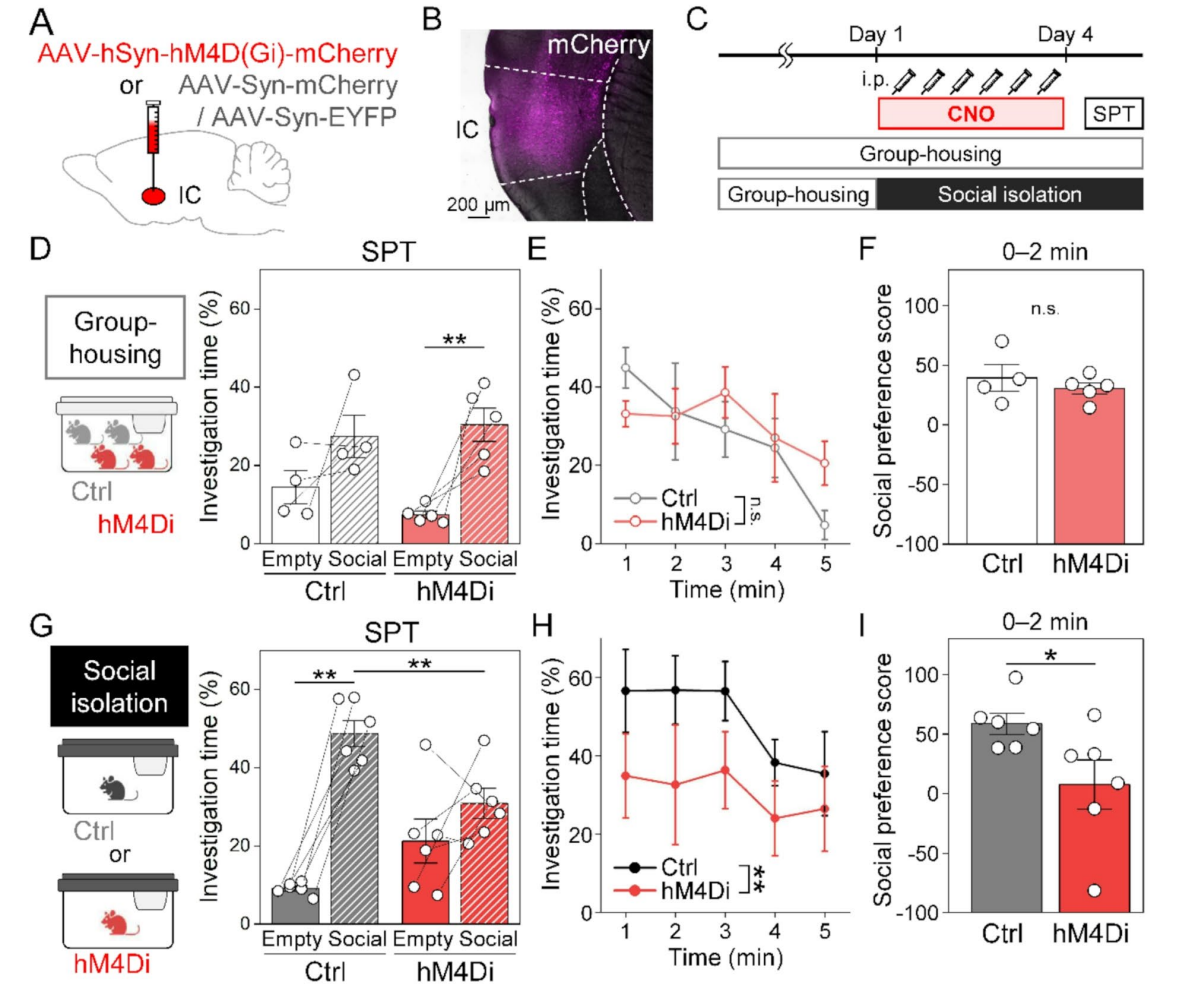
The insular cortex inhibition during social isolation suppressed the enhancement of social preference. (**A**) Schematic diagram of AAV injection in the insular cortex (IC). (**B**) Representative image of hM4D(Gi)-mCherry expression in the insular cortex. (**C**) Experimental schedule for chemogenetic inhibition of the insular cortex during 3 days of group-housing or social isolation. (**D**–**F**) Assessments of social preference following insular cortex inhibition in group-housed mice. Time spent around the cage during the SPT session (**D**, **E**) and the social preference score in the first 0–2 min are shown (**F**). Control (Ctrl), *n* = 4; hM4Di, *n* = 5; two-way repeated measures ANOVA followed by Tukey’s post hoc test (**D**, **E**); two-tailed unpaired *t*-test (**F**); **p < 0.01. (**G**–**I**) Assessments of social preference following insular cortex inhibition in isolated mice. Ctrl, *n* = 6; hM4Di, *n* = 6; two-way repeated measures ANOVA followed by Tukey’s post hoc test (**G**, **H**); two-tailed unpaired *t*-test (**I**); *p < 0.05, **p < 0.01. Data are presented as mean ± S.E.M

To explore the neural mechanisms of isolation-induced rebound social interaction, we focused on the insular cortex, which is known to play a broad role in social and emotion-related behaviors. To investigate whether insular cortex activity modulates social interaction following short-term social isolation, we aimed to suppress its activity during the 3-day isolation period using a DREADD system [21]. Specifically, the inhibitory DREADD hM4Di or, as controls, mCherry or EYFP was expressed in the insular cortex using AAV (Fig. 3A, B). The DREADD agonist clozapine N-oxide (CNO) was administered systemically twice daily during a 3-day period of either group-housing or social isolation period (Fig. 3C).

The inhibition of the insular cortex in group-housed mice did not clearly affect subsequent social preferences (Fig. 3D–F). The investigation time for the social-cue cage was significantly higher than that for the empty cage in the hM4Di group (Fig. 3D, two-way repeated measures ANOVA followed by Tukey’s post hoc test), but there was no notable difference in the social preference score between the control and hM4Di groups (Fig. 3F, two-tailed unpaired *t*-test). On the one hand, regarding the inhibition of the insular cortex in isolated mice, the control group spent significantly more time around the social-cue cage compared to the empty cage, as expected, whereas this feature was absent in the hM4Di group (Fig. 3G, two-way repeated measures ANOVA followed by Tukey’s post hoc test). In addition, the reduction in social interaction in isolated hM4Di mice appeared to persist throughout the entire SPT session—a pattern not observed in group-housed hM4Di mice (Fig. 3E, H, two-way repeated measures ANOVA followed by Tukey’s post hoc test). Furthermore, the social preference score of isolated hM4Di mice was lower than of control mice (Fig. 3I, two-tailed unpaired *t*-test). These results indicate that insular cortex activity during social isolation is required for rebound social interaction following short-term social isolation.

## Discussion

In the present study, we revealed that short-term social isolation enhances rebound social interaction and food consumption. We further demonstrated that activity in the insular cortex during isolation is crucial for modulating subsequent social preference. These findings suggest that the insular cortex plays a vital role in adjusting behavioral strategies in response to changes in the social environment, particularly by mediating social craving following short-term isolation.

Consistent with previous reports, our results showed that short-term social isolation led to an increase in subsequent social interactions (Fig. 1F). Notably, this isolation-induced rebound social interaction was most prominent during the early phase of the social preference test (Fig. 1H, I), which likely reflects an enhanced social valence assigned to conspecifics. The subsequent decline in interaction may indicate a gradual satiation of social craving as social contact is partially fulfilled.

In parallel with the changes in social behavior, short-term isolation also increased food consumption in the home cage (Fig. 2A, B). Interestingly, individuals with higher food intake during isolation also showed higher social preference (Fig. 2D). The correlation between food intake and social interaction may reflect overlapping populations of dopamine neurons in VTA that respond to both food and social stimuli [22]. Moreover, human imaging studies have showed that social cues can evoke craving responses similar to those triggered by food cues after fasting [23]. These findings imply that social and food craving may share common neural substrates influencing motivational behaviors during social isolation.

The insular cortex is involved in integrating external sensory signals and internal physiological states [11]. Previous research has demonstrated that insular cortex neurons modulate their responses to food cues based on hunger and satiety states [24], and this region also plays a crucial role in detecting and processing aversive signals [13, 14, 25, 26]. Given its involvement in state-dependent emotional regulation, the insular cortex seems to be well-positioned to monitor social aversive state and modulate adaptive behavioral responses. Based on this profile, we chose to focus on the insular cortex as a candidate region mediating the motivational and emotional effects of short-term social isolation. While the present study focused on the insular cortex as a first step to determine its causal role in rebound sociability, other brain regions such as the dorsal raphe nucleus and the nucleus accumbens have also been implicated in responses to social isolation [27, 28]. Further studies will be necessary to explore how the insular cortex interacts with other brain regions to orchestrate behavioral adaptation to social deprivation.

Recent studies, such as those by Glangetas et al., indicate that the ablation of insular cortex neurons, which project inter-hemispherically to the contralateral insula cortex, disrupts social preference after short-term social isolation, whereas inhibition of these neurons only during the social preference test does not cause such a reduction [18]. These findings suggest that insular cortex activation during isolation may play a key role in rebound social interaction, though direct verification remained unachieved. In our study, inhibition of the insular cortex during social isolation attenuated subsequent social interactions (Fig. 3G–I), whereas the same manipulation in group-housed mice did not significantly affect subsequent social preference (Fig. 3D–F), indicating that inhibition of insular cortex specifically during the isolation period attenuated social preference. These results provide direct evidence that insular cortex activity during short-term social isolation is essential to trigger the social craving and rebound behavior. Our findings suggest that activation of the insular cortex during isolation enhances the social valence.

Alterations in social valence following social isolation can influence prosocial behaviors that depend on social interactions. While the insular cortex, which is a large structure subdivided along the anterior-posterior and dorso-ventral axes, exhibits both anatomical and functional heterogeneity across its subregions, previous studies have shown that both the anterior and posterior insular cortex are involved in prosocial behaviors [17, 29–31]. In this study, we primarily targeted the medial to posterior insular cortex, where neurons activated during social isolation are more abundantly distributed [18]. Therefore, our findings may also be relevant to the modulation of prosocial behavior. Notably, projection patterns vary across the anterior-posterior and dorsoventral axes of the insular cortex, especially between the anterior and posterior subdivisions involved in emotional signal processing [10, 32]. These differences raise the possibility that distinct subregions of the insular cortex may differentially contribute to prosocial behavior depending on the emotional context.

While our findings highlight the necessity of insular cortex activity during isolation for rebound social interactions, the downstream pathways mediating these effects remain to be elucidated. The insular cortex projects to several limbic and subcortical regions involved in valence processing, including the central amygdala (CeA) and the basolateral amygdala (BLA) [10, 33–35]. Understanding how the insular cortex influences social valence through its downstream targets, particularly under different emotional or motivational conditions, is an important future research.

Although this study focused on male mice, sex differences in behavioral and neuronal responses to social isolation have been reported [28, 36, 37]. In addition, sex differences in electrophysiological properties have also been reported in insular cortex [38]. Whether the insular cortex contributes to isolation-induced rebound sociability in a sex-dependent manner remains an interesting question for future research.

Long-term social isolation is known to induce maladaptive and antisocial behaviors, and linked to activation of the hypothalamic-pituitary-adrenal (HPA) axis and brain-wide changes in neuromodulator expression [7, 39]. In addition, recent studies have revealed the involvement of cortical regions such as the medial prefrontal cortex (mPFC) and the anterior cingulate cortex (ACC) [37, 40]. In contrast, the neural mechanisms underlying rebound social interaction following short-term social isolation have only recently begun to emerge [27, 28, 41, 42], and our study adds to this growing body of evidence. While our study focused on the effects of short-term social isolation, whether the insular cortex also plays a role in responses to long-term isolation remains an important question for future investigation.

In summary, our findings position the insular cortex as a key modulator in the adaptive responses to social deprivation, shaping social valence and motivating behaviors. 

## Electronic supplementary material

Below is the link to the electronic supplementary material.

Supplementary Material 1

## Data Availability

No datasets were generated or analysed during the current study.

## Abbreviations

AAV: Adeno-associated virus
SPT: Social preference test
DREADD: Designer receptors exclusively activated by designer drugs
CNO: Clozapine N-oxide

## References

[1] House JS, Landis KR, Umberson D. Social relationships and health. Science. 1988;241:540–5. 3399889 10.1126/science.3399889

[2] Cacioppo JT, Hawkley LC, Norman GJ. Berntson, social isolation. Ann N Y Acad Sci. 2011;1231:17–22. 21651565 10.1111/j.1749-6632.2011.06028.x PMC3166409

[3] Brandt L, Liu S, Heim C, Heinz A. The effects of social isolation stress and discrimination on mental health. Transl Psychiatry. 2022;12:398. 36130935 10.1038/s41398-022-02178-4 PMC9490697

[4] Li W, et al. Chronic social isolation signals starvation and reduces sleep in Drosophila. Nature. 2021;597:239–44. 34408325 10.1038/s41586-021-03837-0 PMC8429171

[5] Ma XC, et al. Social isolation-induced aggression potentiates anxiety and depressive-like behavior in male mice subjected to unpredictable chronic mild stress. PLoS ONE. 2011;6:e20955. 21698062 10.1371/journal.pone.0020955 PMC3117867

[6] Pibiri F, Nelson M, Guidotti A, Costa E, Pinna G. Decreased corticolimbic allopregnanolone expression during social isolation enhances contextual fear: A model relevant for posttraumatic stress disorder. Proc Natl Acad Sci U S A. 2008;105:5567–72. 18391192 10.1073/pnas.0801853105 PMC2291140

[7] Zelikowsky M, et al. The neuropeptide Tac2 controls a distributed brain state induced by chronic social isolation stress. Cell. 2018;173:1265–79.e1219. 29775595 10.1016/j.cell.2018.03.037 PMC5967263

[8] Niesink RJ, van Ree JM. Short-term isolation increases social interactions of male rats: a parametric analysis. Physiol Behav. 1982;29:819–25. 7156221 10.1016/0031-9384(82)90331-6

[9] Matthews GA, Tye KM. Neural mechanisms of social homeostasis. Ann N Y Acad Sci. 2019;1457:5–25. 30875095 10.1111/nyas.14016 PMC7593988

[10] Gehrlach DA et al. A whole-brain connectivity map of mouse insular cortex. Elife. 2020;9. 10.7554/eLife.55585 PMC7538160 32940600

[11] Gogolla N. The insular cortex. Curr Biol. 2017;27:R580–6. 28633023 10.1016/j.cub.2017.05.010

[12] Juen Z, Villavicencio M, Zuker CS. A neural substrate for short-term taste memories. Neuron. 2024;112:277–87.e274. 37944522 10.1016/j.neuron.2023.10.009

[13] Gehrlach DA, et al. Aversive state processing in the posterior insular cortex. Nat Neurosci. 2019;22:1424–37. 31455886 10.1038/s41593-019-0469-1

[14] Klein AS, Dolensek N, Weiand C, Gogolla N. Fear balance is maintained by bodily feedback to the insular cortex in mice. Science. 2021;374:1010–5. 34793231 10.1126/science.abj8817

[15] Rogers-Carter MM, Christianson JP. An insular view of the social decision-making network. Neurosci Biobehav Rev. 2019;103:119–32. 31194999 10.1016/j.neubiorev.2019.06.005 PMC6699879

[16] Min JY, Park S, Cho J, Huh Y. The anterior insular cortex processes social recognition memory. Sci Rep. 2023;13:10853. 37407809 10.1038/s41598-023-38044-6 PMC10322941

[17] Miura I, et al. Encoding of social exploration by neural ensembles in the insular cortex. PLoS Biol. 2020;18:e3000584. 32956387 10.1371/journal.pbio.3000584 PMC7529241

[18] Glangetas C, et al. A population of Insula neurons encodes for social preference only after acute social isolation in mice. Nat Commun. 2024;15:7142. 39164260 10.1038/s41467-024-51389-4 PMC11336167

[19] Sato M, et al. The lateral parabrachial nucleus is actively involved in the acquisition of fear memory in mice. Mol Brain. 2015;8:22. 25888401 10.1186/s13041-015-0108-z PMC4377188

[20] Conrad KL, Louderback KM, Gessner CP, Winder DG. Stress-induced alterations in anxiety-like behavior and adaptations in plasticity in the bed nucleus of the stria terminalis. Physiol Behav. 2011;104:248–56. 21396387 10.1016/j.physbeh.2011.03.001 PMC3118978

[21] Armbruster BN, Li X, Pausch MH, Herlitze S, Roth BL. Evolving the lock to fit the key to create a family of G protein-coupled receptors potently activated by an inert ligand. Proc Natl Acad Sci U S A. 2007;104:5163–8. 17360345 10.1073/pnas.0700293104 PMC1829280

[22] Willmore L, et al. Overlapping representations of food and social stimuli in mouse VTA dopamine neurons. Neuron. 2023;111:3541–53.e3548. 37657441 10.1016/j.neuron.2023.08.003 PMC11672631

[23] Tomova L, et al. Acute social isolation evokes midbrain craving responses similar to hunger. Nat Neurosci. 2020;23:1597–605. 33230328 10.1038/s41593-020-00742-z PMC8580014

[24] Livneh Y, et al. Homeostatic circuits selectively gate food cue responses in insular cortex. Nature. 2017;546:611–6. 28614299 10.1038/nature22375 PMC5577930

[25] Wang Q, et al. Insular cortical circuits as an executive gateway to Decipher threat or extinction memory via distinct subcortical pathways. Nat Commun. 2022;13:5540. 36130959 10.1038/s41467-022-33241-9 PMC9492683

[26] Labrakakis C. The role of the insular cortex in pain. Int J Mol Sci. 2023;24. 10.3390/ijms24065736 PMC10056254 36982807

[27] Matthews GA, et al. Dorsal Raphe dopamine neurons represent the experience of social isolation. Cell. 2016;164:617–31. 26871628 10.1016/j.cell.2015.12.040 PMC4752823

[28] Choi JE, et al. Synaptic ensembles between Raphe and D(1)R-containing accumbens shell neurons underlie postisolation sociability in males. Sci Adv. 2022;8:eabo7527. 36223467 10.1126/sciadv.abo7527 PMC9555785

[29] Rogers-Carter MM, et al. Insular cortex mediates approach and avoidance responses to social affective stimuli. Nat Neurosci. 2018;21:404–14. 29379116 10.1038/s41593-018-0071-y PMC6051351

[30] Rogers-Carter MM, Djerdjaj A, Gribbons KB, Varela JA, Christianson JP. Insular cortex projections to nucleus accumbens core mediate social approach to stressed juvenile rats. J Neurosci. 2019;39:8717–29. 31591155 10.1523/JNEUROSCI.0316-19.2019 PMC6820210

[31] Cox SS, et al. The role of the anterior insular during targeted helping behavior in male rats. Sci Rep. 2022;12:3315. 35228625 10.1038/s41598-022-07365-3 PMC8885669

[32] Zhang R, Deng H, Xiao X. The insular cortex: an interface between sensation, emotion and cognition. Neurosci Bull. 2024;40:1763–73. 38722464 10.1007/s12264-024-01211-4 PMC11607240

[33] Ponserre M, Peters C, Fermani F, Conzelmann KK, Klein R. The Insula cortex contacts distinct output streams of the central amygdala. J Neurosci. 2020;40:8870–82. 33051345 10.1523/JNEUROSCI.0567-20.2020 PMC7659460

[34] Nicolas C, et al. Linking emotional valence and anxiety in a mouse insula-amygdala circuit. Nat Commun. 2023;14:5073. 37604802 10.1038/s41467-023-40517-1 PMC10442438

[35] Takemoto M, Kato S, Kobayashi K, Song WJ. Dissection of insular cortex layer 5 reveals two sublayers with opposing modulatory roles in appetitive drinking behavior. iScience. 2023;26:106985. 37378339 10.1016/j.isci.2023.106985 PMC10291511

[36] Senst L, Baimoukhametova D, Sterley TL, Bains JS. Sexually dimorphic neuronal responses to social isolation. Elife. 2016;5. 10.7554/eLife.18726 PMC5059136 27725087

[37] Tan T, et al. Neural circuits and activity dynamics underlying sex-specific effects of chronic social isolation stress. Cell Rep. 2021;34:108874. 33761364 10.1016/j.celrep.2021.108874

[38] Iezzi D, Cáceres-Rodríguez A, Strauss B, Chavis P, Manzoni OJ. Sexual differences in neuronal and synaptic properties across subregions of the mouse insular cortex. Biol Sex Differ. 2024;15:29. 38561860 10.1186/s13293-024-00593-4 PMC10983634

[39] Hawkley LC, Cole SW, Capitanio JP, Norman GJ, Cacioppo JT. Effects of social isolation on glucocorticoid regulation in social mammals. Horm Behav. 2012;62:314–23. 22663934 10.1016/j.yhbeh.2012.05.011 PMC3449017

[40] Guo B, et al. CB1R dysfunction of inhibitory synapses in the ACC drives chronic social isolation stress-induced social impairments in male mice. Neuron. 2024;112:441–57.e446. 37992714 10.1016/j.neuron.2023.10.027

[41] Lee CR et al. Separable dorsal raphe dopamine projections mediate the facets of loneliness-like state. bioRxiv. 2025.2002.2003.636224 (2025). 10.7554/eLife.105955 PMC12316461 40747785

[42] Liu D, et al. A hypothalamic circuit underlying the dynamic control of social homeostasis. Nature. 2025;640:1000–10. 40011768 10.1038/s41586-025-08617-8 PMC12018270

