# Supplementary material for: Modulation of social valence by insular cortex activity during acute social isolation in mice
Source: Mol Brain. 2025 Jul 15;18:62. doi: 10.1186/s13041-025-01236-4 (PMC12265204; doi:10.1186/s13041-025-01236-4)
Supplement: Supplementary file 1 — Supplementary Material 1 [file 13041_2025_1236_MOESM1_ESM.pdf]

Additional File 1

Supplementary Table 1. Statistical summary

Figure 1

Panel	Experiment	Sample size	Statical Test	Comparison	Statistic	P-values	Post-hoc	P-values
B	Open field test_Total distance	Grouped (n=8), Isolated (n=8)	Two-tailed unpaired t-test		t=-0.16627, df=14	0.87032		
C	Open field test_Center time	Grouped (n=8), Isolated (n=8)	Two-tailed unpaired t-test		t=1.0958, df=14	0.29166		
F	Social preference test_Before	Grouped (n=8), Isolated (n=8)	Two-way RM ANOVA	Factor 1: Group (Grouped vs Isolated)	F(1,14) = 0.44675	0.51475	(Grouped) Cage 1 - Cage 2	0.567820
				Factor 2: Cue (Cage 1 vs Cage 2)	F(1,14) = 0.00651	0.93683	(Isolated) Cage 1 - Cage 2	0.495900
				Interaction (F1 x F2)	F(1,14) = 0.82461	0.3792	(Cage 1) Grouped - Isolated	0.279410
							(Cage 2) Grouped - Isolated	0.692960
	Social preference test_SPT	Grouped (n=8), Isolated (n=8)	Two-way RM ANOVA, Tukey's post hoc	Factor 1: Group (Grouped vs Isolated)	F(1,14) = 4.09314	0.06259	(Grouped) Empty - Social	0.000232
				Factor 2: Cue (Empty vs Social)	F(1,14) = 72.47553	<0.0001	(Isolated) Empty - Social	<0.0001
				Interaction (F1 x F2)	F(1,14) = 2.47685	0.13785	(Empty) Grouped - Isolated	0.873130
							(Social) Grouped - Isolated	0.024530
H	Social preference test_binning 1 min	Grouped (n=8), Isolated (n=8)	Two-way RM ANOVA, Tukey's post hoc	Factor 1: Group (Grouped vs Isolated)	F(1,14) = 3.88584	0.06879	Grouped - Isolated	
				Factor 2: Time	F(4,56) = 5.19495	0.00125	1min	0.259760
				Interaction (F1 x F2)	F(4,56) = 1.59965	0.18716	2min	0.010300
							3min	0.431800
							4min	0.756790
							5min	0.503310
I	Social preference test_0–2 min	Grouped (n=8), Isolated (n=8)	Two-tailed unpaired t-test		t=-2.93434, df=14	0.01088		

Figure 2

Panel	Experiment	Sample size	Statical Test	Comparison	Statistic	P-values	Post-hoc	P-values
A	3 days food intake	Grouped (n=3), Isolated (n=13)	Two-tailed unpaired t-test		t=-3.24868, df=14	0.00583		
B	The ratio of food intake	n=13	one-sample t-test		t=7.74175, df=12	<0.0001		
C	The ratio of body weight	Grouped (n=13), Isolated (n=13)	Two-tailed unpaired t-test		t=-1.22925, df=24	0.2309		
D	Correlation between food intake and social preference score	Isolated (n=13)	linear regression (Pearson)		r=0.67054	0.06877		

Figure 3

Panel	Experiment	Sample size	Statical Test	Comparison	Statistic	P-values	Post-hoc	P-values
D	Social preference test_SPT	Ctrl (n=4), hM4Di (n=5)	Two-way RM ANOVA, Tukey's post hoc	Factor 1: Group (Ctrl vs hM4Di)	F(1,7) = 0.45895	0.51987	(Ctrl) Empty - Social	0.140630
				Factor 2: Cue (Empty vs Social)	F(1,7) = 15.20511	0.0059	(hM4Di) Empty - Social	0.006520
				Interaction (F1 x F2)	F(1,7) = 1.19208	0.31106	(Empty) Ctrl - hM4Di	0.507550
							(Social) Ctrl - hM4Di	0.925870
E	Social preference test_binning 1 min	Ctrl (n=4), hM4Di (n=5)	Two-way RM ANOVA, Tukey's post hoc	Factor 1: Group (Ctrl vs hM4Di)	F(1,7) = 0.19227	0.67425		
				Factor 2: Time	F(4,28) = 4.95478	0.00379		
				Interaction (F1 x F2)	F(4,28) = 1.30194	0.29336		
F	Social preference test_0–2 min	Ctrl (n=4), hM4Di (n=5)	Two-tailed unpaired t-test		t=0.79094, df=7	0.45494		
G	Social preference test_SPT	Ctrl (n=6), hM4Di (n=6)	Two-way RM ANOVA, Tukey's post hoc	Factor 1: Group (Ctrl vs hM4Di)	F(1,10) = 0.83617	0.38201	(Ctrl) Empty - Social	<0.0001
				Factor 2: Cue (Empty vs Social)	F(1,10) = 31.80877	0.000215	(hM4Di) Empty - Social	0.150850
				Interaction (F1 x F2)	F(1,10) = 11.83298	0.00633	(Empty) Ctrl - hM4Di	0.047030
							(Social) Ctrl - hM4Di	0.007630
H	Social preference test_binning 1 min	Ctrl (n=6), hM4Di (n=6)	Two-way RM ANOVA, Tukey's post hoc	Factor 1: Group (Ctrl vs hM4Di)	F(1,10) = 12.43001	0.00549		
				Factor 2: Time	F(4,40) = 1.1144	0.36322		
				Interaction (F1 x F2)	F(4,40) = 0.16496	0.9549		
I	Social preference test_0–2 min	Ctrl (n=4), hM4Di (n=5)	Two-tailed unpaired t-test		t=2.26389, df=10	0.04706		
